# Analysis of the Hypoxic Response in a Mouse Cortical Collecting Duct-Derived Cell Line Suggests That Esrra Is Partially Involved in Hif1α-Mediated Hypoxia-Inducible Gene Expression in mCCD_cl1_ Cells

**DOI:** 10.3390/ijms23137262

**Published:** 2022-06-30

**Authors:** Anna Keppner, Darko Maric, Ilaria Maria Christina Orlando, Laurent Falquet, Edith Hummler, David Hoogewijs

**Affiliations:** 1 Section of Medicine, Department of Endocrinology, Metabolism and Cardiovascular System (EMC), Faculty of Science and Medicine, University of Fribourg, Chemin du Musée 5, CH-1700 Fribourg, Switzerland; anna.keppner@unifr.ch (A.K.); darko.maric@unifr.ch (D.M.); ilaria.orlando@unifr.ch (I.M.C.O.); 2 National Center of Competence in Research Kidney Control of Homeostasis (NCCR Kidney.CH), University of Zurich, CH-8006 Zürich, Switzerland; 3 Section of Science, Department of Biology, Faculty of Science and Medicine, University of Fribourg, CH-1700 Fribourg, Switzerland; laurent.falquet@unifr.ch; 4 Department of Biomedical Sciences, University of Lausanne, CH-1011 Lausanne, Switzerland; edith.hummler@unil.ch

**Keywords:** hypoxia, collecting duct, Hif, oxygen, kidney

## Abstract

The kidney is strongly dependent on a continuous oxygen supply, and is conversely highly sensitive to hypoxia. Controlled oxygen gradients are essential for renal control of solutes and urine-concentrating mechanisms, which also depend on various hormones including aldosterone. The cortical collecting duct (CCD) is part of the aldosterone-sensitive distal nephron and possesses a key function in fine-tuned distal salt handling. It is well known that aldosterone is consistently decreased upon hypoxia. Furthermore, a recent study reported a hypoxia-dependent down-regulation of sodium currents within CCD cells. We thus investigated the possibility that cells from the cortical collecting duct are responsive to hypoxia, using the mouse cortical collecting duct cell line mCCD_cl1_ as a model. By analyzing the hypoxia-dependent transcriptome of mCCD_cl1_ cells, we found a large number of differentially-expressed genes (3086 in total logFC< −1 or >1) following 24 h of hypoxic conditions (0.2% O_2_). A gene ontology analysis of the differentially-regulated pathways revealed a strong decrease in oxygen-linked processes such as ATP metabolic functions, oxidative phosphorylation, and cellular and aerobic respiration, while pathways associated with hypoxic responses were robustly increased. The most pronounced regulated genes were confirmed by RT-qPCR. The low expression levels of *Epas1* under both normoxic and hypoxic conditions suggest that Hif-1α, rather than Hif-2α, mediates the hypoxic response in mCCD_cl1_ cells. Accordingly, we generated shRNA-mediated Hif-1α knockdown cells and found Hif-1α to be responsible for the hypoxic induction of established hypoxically-induced genes. Interestingly, we could show that following shRNA-mediated knockdown of Esrra, Hif-1α protein levels were unaffected, but the gene expression levels of *Egln3* and *Serpine1* were significantly reduced, indicating that Esrra might contribute to the hypoxia-mediated expression of these and possibly other genes. Collectively, mCCD_cl1_ cells display a broad response to hypoxia and represent an adequate cellular model to study additional factors regulating the response to hypoxia.

## 1. Introduction

The renal blood supply represents one of the highest in the body, with a blood flow that is estimated at 22% of cardiac output [1]. This high flow rate is essential for kidney-mediated elimination of metabolic waste products via the urine. Despite this extremely high blood supply, it is estimated that only 10% of the transported oxygen is sufficient to fulfill the kidneys’ metabolic demands [1]. Strikingly, the oxygen supply to the kidney is highly variable, whereby the majority is delivered to the renal cortex, and only scarce amounts to the renal medulla, thereby creating a steep cortico-medullary oxygen gradient, which further leads to a hypoxic environment within the loop of Henle and the inner medulla [2,3]. This is of physiological importance, since this gradient is essential for the countercurrent exchange of solutes and the maintenance of osmotic gradients to optimally concentrate the urine at the level of the thick ascending limb [4]. Despite the importance of the corticomedullary oxygen gradient, the kidney is highly sensitive to hypoxia, which is an important pathological aspect in various diseases such as chronic kidney disease (CKD) and acute kidney injury [5,6]. 

The key component of the cellular adaptation to low oxygen is the hypoxia-inducible factor (HIF) pathway, which relies on the transcriptional complex formed by the oxygen-labile nucleocytoplasmic HIFα (HIF-1α and HIF-2α) and nuclear HIFβ transcription factors. The constitutively-expressed HIFα subunit is degraded in the presence of sufficient oxygen following its hydroxylation at conserved proline residues by prolyl hydroxylase domain enzymes (PHD1, PHD2, and PHD3, encoded by the *Egln2*, *Egln1*, and *Egln3* genes, respectively), enabling its recognition by the VHL E3 ubiquitin ligase and targeting for proteasomal degradation. When oxygen levels are low, the activity of PHD enzymes is strongly reduced, since their enzymatic activity depends on oxygen as a substrate, thereby allowing HIFα stabilization, nuclear translocation, and heterodimerization with HIFβ. The active heterodimer is then able to bind to specific hypoxia response elements (HRE) and to induce the transcriptional activation of approximately 1000 target genes per cell type. Notably, PHD2 and PHD3, but not PHD1, are HIF target genes induced under hypoxic conditions [7,8,9].

The kidney is responsible for maintaining blood osmolality and fluid balance through the tight regulation of water and solute reabsorption. To achieve this function, the renal tubules are controlled by various hormones and other factors that will influence fluid and sodium reabsorption. Aldosterone is a key mineralocorticoid hormone whose secretion triggers sodium reabsorption via the epithelial sodium channel (ENaC) and potassium secretion via the renal outer medullary K+ channel within the aldosterone-sensitive distal nephron (ASDN), which includes the distal convoluted tubule, the connecting tubule, and the cortical collecting duct (CCD) [10]. Interestingly, for decades, numerous studies have consistently reported a decrease in aldosterone secretion following hypoxia [11,12,13,14,15,16]. A recent study by Dizin and colleagues [17] that was conducted in the cortical collecting duct mpkCCD_cl4_ cell line demonstrated that the activation of the HIF pathway following hypoxia or dimethyloxalylglycine treatment leads to decreased sodium transport and ENaC expression. Similar results were obtained following the induction of reactive oxygen species (ROS). This study further confirmed that the HIF pathway controls ENaC, using kidney tubule-specific inducible Hif-1α knockout mice which displayed increased ENaC protein expression following hypoxia. The effects of HIF-1α on ENaC and Na^+^, K^+^-ATPase are likely indirect however, since no putative HIF binding site was found in the proximal regulatory regions of their genes. The authors suggested that the inhibition of ENaC-mediated sodium transport may prevent increased ATP—and thus oxygen—consumption via the basolateral Na^+^, K^+^-ATPase under hypoxic conditions [17]. 

Considering the intriguing relation that seems present between aldosterone, the HIF pathway, and ENaC, we hypothesized that cortical collecting duct cells might be responsive to hypoxia overall, and that these cells may serve as a physiological model to study hypoxia-related cellular processes. In this study, we therefore analyze the mouse cortical collecting duct cell line mCCD_cl1_, a polarized epithelial cell line generated by Gaeggeler and coworkers, which have been described to exhibit both aldosterone- and corticosterone-dependent sodium transport [18]. Our results demonstrate that mCCD_cl1_ cells respond to hypoxia by up-regulating Hif-1 target genes. Additionally, we could validate mCCD_cl1_ cells as a cellular model for potentially protective genes against hypoxia-driven kidney injury by analyzing estrogen-related receptor alpha (ESSRA), a transcription factor that was recently identified as protective against kidney injury in proximal tubule cells [19]. 

## 2. Results

### 2.1. RNAseq of mCCD_cl1_ Cells Reveals Enrichment of Hypoxic Genes following 24 h of Hypoxia

**Figure 1:**
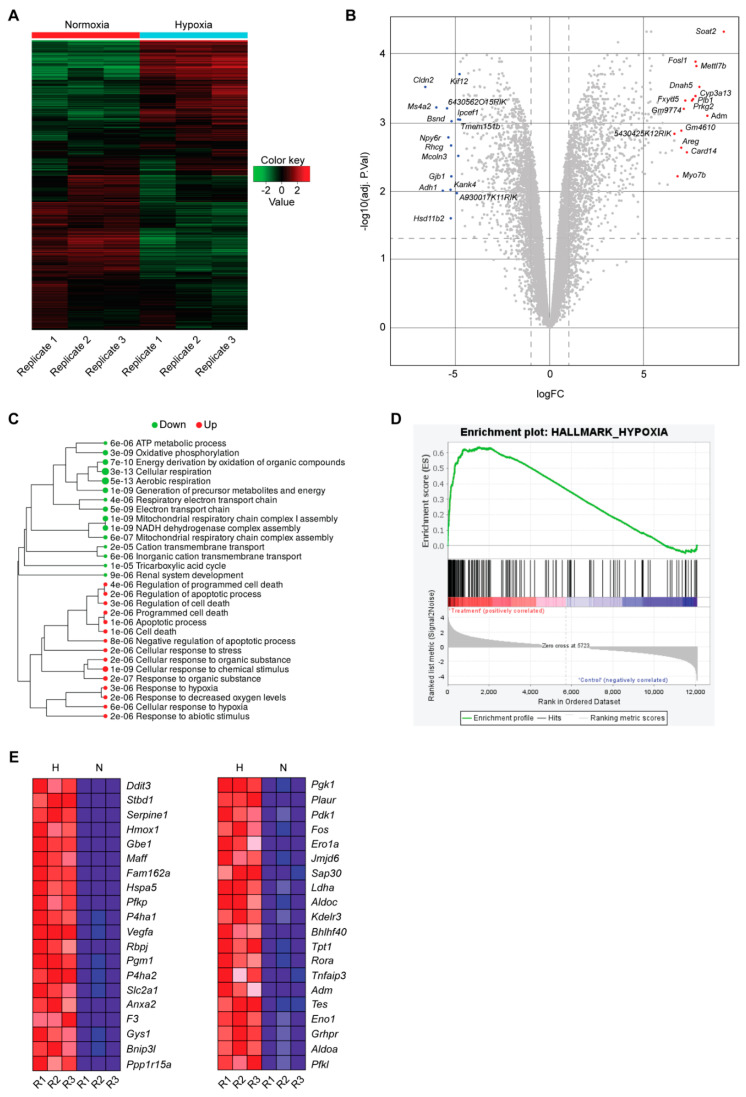
RNAseq of mCCD_cl1_ cells following 24 h of normoxia (21% O_2_) or 24 h of hypoxia (0.2% O_2_). (**A**) Heatmap of the 1000 most up- or down-regulated genes following normoxia or hypoxia (*n* = 3 per condition). Green indicates down-regulated genes, and red up-regulated genes. (**B**) Volcano plot of differentially expressed genes in mCCD_cl1_ cells following 24 h hypoxia: the x-axis represents the log2 fold change, and the y-axis represents log10 of the adjusted *p*-value. The top 15 up- and down-regulated genes are indicated in red and blue, respectively. (**C**) Gene ontology analysis of significantly up- (red) and down-regulated (green) pathways following 24 h hypoxia. Adjusted *p*-values are indicated, with a false discovery rate (FDR) of 0.1. (**D**) Enrichment plot of genes associated with the GO hallmark term “hypoxia” (normoxia “control”, and hypoxia “treatment”). (**E**) Top 40 genes (1–20 left column, 21–40 right column) within the GO hallmark term “hypoxia” in mCCD_cl1_ cells enriched following hypoxia (H) relative to normoxia (N). The three replicates are indicated (R1, R2, and R3).

**Figure 2:**
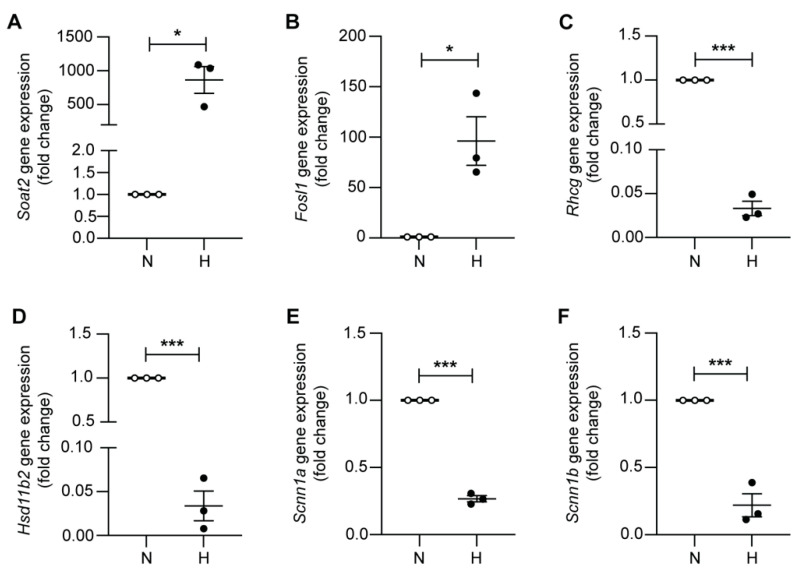
RNAseq validation of differentially-regulated genes. Relative mRNA expression levels of (**A**) *Soat2*, (**B**) *Fosl1*, (**C**) *Rhcg*, (**D**) *HSD11b2*, (**E**) *Scnn1a* (αENaC), and (**F**) *Scnn1b* (βENaC) in mCCD_cl1_ cells following 24 h of normoxia (N) or hypoxia (H) (*n* = 3 per condition). * *p* < 0.05, *** *p* < 0.001.

To determine whether mCCD_cl1_ cells are able to elicit a robust hypoxic response, we subjected the cells to normoxia (21% O_2_) or to 24 h of hypoxia (0.2% O_2_), and analyzed their transcriptomes by RNAseq. No significant impact on cell viability could be observed after 24 h of 0.2% O_2_, as also seen for other cell lines [20]. The results revealed 1679 up-regulated genes (logFC > 1), and 1407 down-regulated genes (logFC < −1) following 24 h hypoxia (Figure 1A,B, Table S1). A gene ontology analysis of the differentially-regulated pathways further revealed a strong decrease in oxygen-linked processes such as ATP metabolic functions, oxidative phosphorylation, and cellular and aerobic respiration, while pathways associated with hypoxic responses were robustly increased (Figure 1C), as also illustrated by k-Means enrichment analysis (Figure S1). Analysis of the gene enrichment for the hallmark hypoxia (Figure 1D) confirmed the up-regulation of Hif-target genes such as *Serpine1* and *Vegfa*, and the Hif-1α interactor *Fam162a*, but also genes associated with glycolytic processes including *Pfkp* and *Gbe1* (Figure 1E). Furthermore, specific enrichment analysis for the Hif-1α pathway was observed in mCCD_cl1_ cells following hypoxia (Figure S2). Consistent with the restricted renal expression pattern of Hif-2α in endothelial cells and interstitial fibroblasts, the expression levels of *Epas1* were barely detectable in mCCD_cl1_ cells under both normoxic and hypoxic conditions, suggesting that Hif-1 rather than Hif-2 mediates the hypoxic response in mCCD_cl1_ cells. To validate the RNAseq data, we assessed the gene expression levels of *Soat2* and *Fosl1,* which ranked among the top 15 most up-regulated genes following hypoxia, and conversely *Rhcg* and *Hsd11b2* (encoding the aldosterone synthase) which ranked among the 15 most down-regulated genes. RT-qPCR results confirmed that these genes were indeed significantly regulated by hypoxia (Figure 2). Furthermore, the mRNA expression levels of *Scnn1a* (αENaC) and *Scnn1b* (βENaC) were significantly reduced following hypoxia, consistent with the RNAseq data, confirming the observations of Dizin and colleagues [17].

### 2.2. mCCD_cl1_ Cells Display Hif-1α-Mediated Regulation of Hypoxic Genes

**Figure 3:**
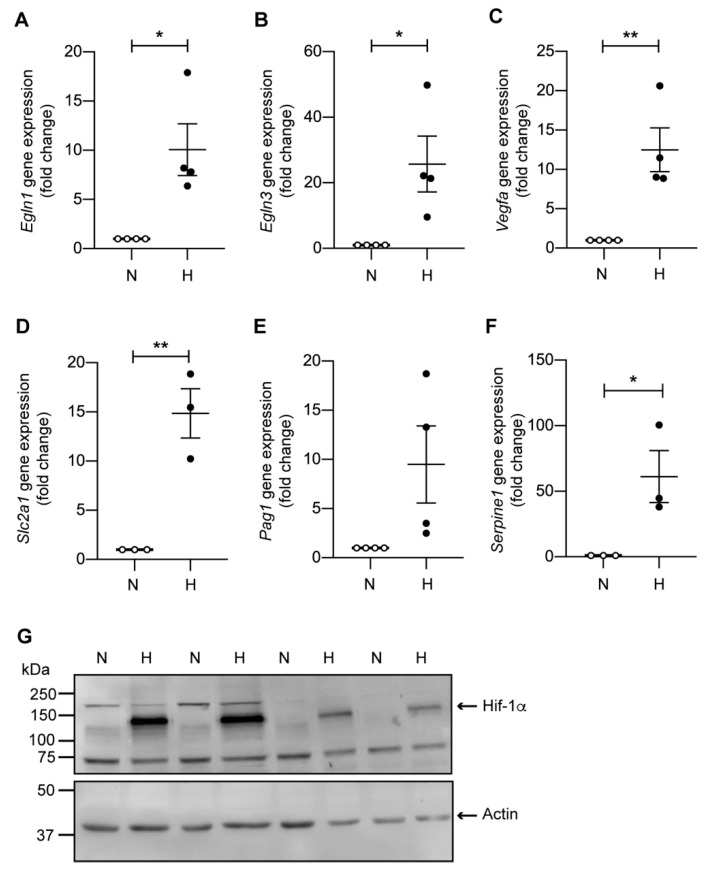
mCCD_cl1_ cells display up-regulation of hypoxic markers. Relative mRNA expression levels of (**A**) *Egln1*, (**B**) *Egln3*, (**C**) *Vegfa*, (**D**) *Slc2a1*, (**E**) *Pag1*, and (**F**) *Serpine1* in mCCDcl1 cells in normoxia (N) and following 24 h of hypoxia (H) (*n* = 4 per condition in (**A**–**C**,**E**), *n* = 3 per condition in (**D**,**F**) secondary to insufficient RNA for one sample). (**G**) Representative immunoblots of Hif-1α in protein lysates of mCCD_cl1_ cells under normoxia (N) or following 24 h hypoxia (H). β-actin was used as loading control. * *p* < 0.05, ** *p* < 0.01.

**Figure 4:**
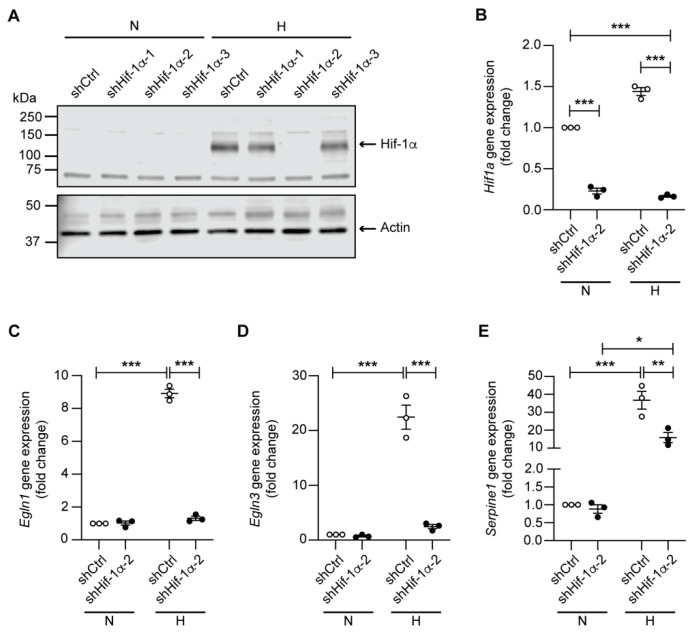
shRNA-mediated knockdown of Hif-1α leads to a down-regulation of hypoxic marker genes. (**A**) Representative immunoblot of Hif-1α in mCCD_cl1_ cells transduced with a control shRNA (shCtrl) and three different shRNAs targeting Hif-1α (shHif-1α-1-3) in normoxia (N) or following 24 h of hypoxia (H). β-actin was used as loading control. Relative mRNA expression levels of (**B**) *Hif1a*, (**C**) *Egln1*, (**D**) *Egln3*, and (**E**) *Serpine1* in mCCD_cl1_ cells transduced with a control shRNA (shCtrl) and a shRNA targeting Hif-1α (shHif-1α-2) in normoxia (N) or following 24 h of hypoxia (H) (*n* = 3 per condition). * *p* < 0.05, ** *p* < 0.01, *** *p* < 0.001.

We next investigated the expression of genes directly regulated by Hif-1α. Following 24 h of hypoxia, mCCD_cl1_ cells displayed a strong and robust up-regulation of *Egln1*, *Egln3*, *Vegfa*, *Slc2a1* (Glut1), *Pag1,* and *Serpine1* (Figure 3A–F), whereby the protein expression of Hif-1α itself was consistently enhanced in all analyzed replicates under hypoxic conditions (Figure 3G). We next aimed to knock down Hif-1α in mCCD_cl1_ cells using lentiviral-based short hairpin RNA (shRNA) transduction. Three different shRNA sequences targeting Hif-1α were tested, from which one (shHif-1α-2) led to a reduction of 80–85% of *Hif1a* expression as compared to the control shRNA under both normoxic and hypoxic conditions (Figure 4A,B). Efficient knockdown of Hif-1α suppressed the hypoxia-dependent up-regulation of *Egln1* and *Egln3*, and also induced a significant reduction in *Serpine1* expression under hypoxic conditions (Figure 4C–E).

### 2.3. Knockdown of Esrra Leads to Reduced Egln3 and Serpine1 Gene Expression following 24 h of Hypoxia

**Figure 5:**
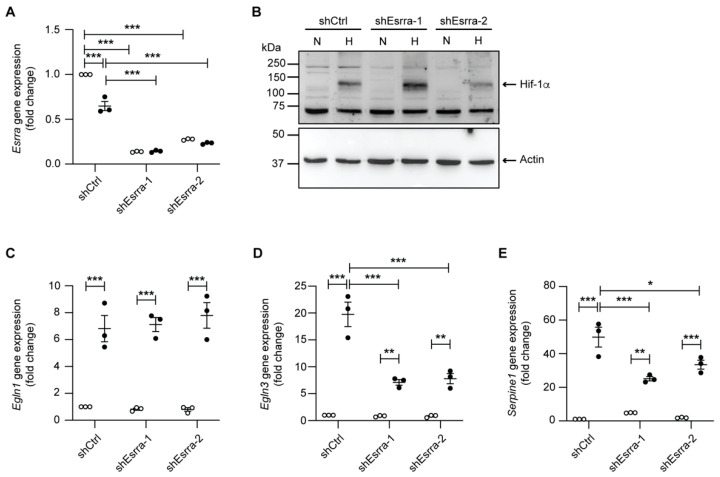
shRNA-mediated knockdown of ESRRA leads to reduced *Egln3* and *Serpine1* levels independently of Hif-1α. (**A**) Relative mRNA expression levels of *Esrra* in mCCD_cl1_ cells transduced with control shRNA (shCtrl) and two different shRNAs targeting Esrra (shEsrra-1 and shEsrra-2) in normoxia (N) or following 24 h of hypoxia (H) (*n* = 3 per condition). (**B**) Representative immunoblot of Hif-1α in mCCD_cl1_ cells transduced with control shRNA (shCtrl) and two different shRNAs targeting Esrra (shEsrra-1 and shEsrra-2) in normoxia (N) or following 24 h of hypoxia (H). β-actin was used as a loading control. Relative mRNA expression levels of (**C**) *Egln1*, (**D**) *Egln3,* and (**E**) *Serpine1* in mCCD_cl1_ cells transduced with control shRNA (shCtrl) and two different shRNAs targeting Esrra (shEsrra-1 and shEsrra-2) in normoxia (N) or following 24 h of hypoxia (H) (*n* = 3 per condition). * *p* < 0.05, ** *p* < 0.01, *** *p* < 0.001.

Comparative chromatin immunoprecipitation (ChIP)-sequencing analysis in human cell lines between HIF-1α, HIF-2α, and HIF-β, and potential co-factors of HIF-mediated transcriptional responses based on the publicly-available ENCODE data revealed the estrogen-related receptor alpha (ESRRA) as a candidate transcription factor (Figure S3). To test whether ESRRA represents a transcriptional regulator of hypoxia-dependent transcription of target genes in mCCD_cl1_ cells, we generated shRNA-mediated knockdown cells. Both shRNAs targeting Esrra led to a significant reduction in its mRNA expression (87% reduction for shEsrra-1, and 73% reduction for shEsrra-2) under normoxic conditions (Figure 5A). After 24 h of hypoxia, *Esrra* expression levels were reduced in control shRNA cells (Figure 5A), consistent with the RNAseq which indicated a moderate down-regulation of *Esrra*. Interestingly, despite unchanged Hif-1α protein expression levels and no change in *Egln1* mRNA expression levels (Figure 5B,C), the knockdown of Esrra significantly reduced gene expression levels of *Egln3* and *Serpine1* after 24 h of hypoxia (Figure 5D,E). Consistent with this observation, ChIP-seq data from human cell lines further illustrate a broad overlap between HIF and ESRRA binding sites at the SERPINE1 locus (Figure S4).

## 3. Discussion

In this study, we provided evidence that mCCD_cl1_ cells respond to hypoxia in a Hif-1-dependent manner. Along with the study of Dizin and colleagues, who showed that activation of the Hif pathway leads to a reduction in ENaC and of Na^+^,K^+^-ATPase expression in mpkCCD_cl4_ cells [17], this raises the possibility that cells of the aldosterone-sensitive distal nephron are responsive to hypoxia overall. It is well described that the glomeruli, proximal tubules, and thick ascending limb of Henle’s loop are the most sensitive to hypoxia given their high energy consumption rates [21]; however, it is intriguing that aldosterone levels decrease following acute hypoxia, while most studies focusing on renin secretion have reported a rapid rise in plasmatic and renal renin levels, leading to a temporary uncoupling between aldosterone and renin [22,23]. After a prolonged exposure to hypoxia, renin levels decrease below the baseline, suggesting an adaptive and transitional reaction [24]. Interestingly, the rise in renin is observable in vivo but not in primary cultured juxtaglomerular cells [22,23]. Thus, the ASDN might play a pivotal role in the early adaptation to hypoxia.

It is highly likely that the decrease in aldosterone, together with increased natriuresis upon hypoxia, is an energy-sparing mechanism to reduce Na^+^,K^+^-ATPase-mediated ATP consumption within the cortical collecting duct, in order to protect the more sensitive parts of the nephron from insufficient energy supply [17]. In line with this hypothesis, the levels of the Na^+^,K^+^-ATPase were also reduced in the transcriptome of mCCD_cl1_ cells following hypoxia (Table S1). Accordingly, our transcriptome analysis revealed the up-regulation of various HIF-1α target genes linked to anaerobic glucose metabolism including platelet-type phosphofructokinase (*Pfkp*) [25,26], glycogen synthase 1 (*Gys1*), and the related 1,4-alpha-glucan branching enzyme (*Gbe1*) [27] (Figure 1). We could further demonstrate up-regulation at the mRNA level of the glucose transporter 1 (Glut1, *Slc2a1*) which was equally enriched in the GSE analysis (Figure 1). This result is consistent with previously published studies showing that, upon intracellular ATP reduction following oxygen deprivation, HIF-1α promotes the expression and translocation of Glut1 to the cellular membrane, contributing to increased glucose uptake and the restoration of energy production in various cell lines including adipose-derived stem cells, human umbilical vein endothelial cells, and alveolar epithelial cells [28,29,30]. The transport of vesicles carrying Glut1 to the membrane involves the cell cytoskeleton and microtubules, and is promoted by several kinases such as protein kinase C, casein kinase substrate 3, AMP kinase, protein kinase B, and phosphoinositide 3-kinase. We could further validate the up-regulation of other notable Hif-1α target genes, such as the vascular endothelial growth factor A (*Vegfa*), serpin family E member 1 (*Serpine1*), and the more recently characterized HIF target phosphoprotein membrane anchor with glycosphingolipid microdomains 1 (*Pag1*), which all have a link with cancer progression and the tumor microenvironment [31,32,33,34,35]. These hypoxia-mediated regulations are intriguing, since mCCD_cl1_ cells are not cancerous cells, suggesting that despite their association with cancer, these genes participate in broader cellular processes. Thus, mCCD_cl1_ may further represent an interesting cell model capable of phenocopying healthy physiological cellular hypoxic responses.

In opposition with routinely-used cell lines in hypoxia research, we could not detect *Epas1* (Hif-2α) in the RNAseq data from mCCD_cl1_ cells under either normoxic and hypoxic conditions, nor on a protein level under hypoxic conditions (data not shown), suggesting a lack of *Epas1* expression. Furthermore, the hypoxic response of several canonical HIF target genes was almost fully abrogated in shHif-1α cells, demonstrating the lack of redundancy of the HIF response in these cells. This finding is in line with the generally accepted view that Hif-2α is expressed in fibroblasts and some peritubular endothelial cells but not in tubular cells of the nephron, whereas Hif-1α expression can be detected predominantly in tubular epithelial cells [36,37]. It is noteworthy that the targeted deletion of VHL in renin-producing cells leads to an accumulation of Hif-1α, but not Hif-2α, in collecting duct cells in adult mouse kidneys [38], further supporting a possible Hif-1α function in this part of the nephron.

Despite a similar conserved consensus DNA binding site, HIF-1 and HIF-2 bind different but overlapping sets of sites in chromatin and transactivate only partially overlapping patterns of gene expression. It has been suggested that both the DNA-binding and heterodimerization domain as well as the transactivation domain contribute to HIFα isoform selectivity and accordingly target gene selectivity [39]. Correspondingly, Ortmann and coworkers have recently demonstrated HIF-selective recruitment through its PAS-B domain of the histone 3 lysine 4 (H3K4) methyltransferase SET1B, resulting in site-specific H3K4 methylation and rapid activation of HIF target genes [40]. Furthermore, a recent pan-genomic HIF isoform distribution study has suggested that interactions with other transcription factors might mediate the binding specificity of HIF isoforms [41]. Consistent with our findings, this study proposed an enrichment of ESRRA binding sites in normoxic HepG2 cells at canonical HIF-1 and HIF-2 sites in the genome of HepG2 cells exposed to hypoxic conditions. Surprisingly, Esrra knockdown resulted in reduced hypoxic induction of *Serpine1*, a canonical HIF-2 specific target gene. Given the absence of *Epas1* expression in mCCD_cl1_ cells, it remains to be determined whether Esrra indeed regulates a broader subset of hypoxia-induced transcripts and possibly confers HIFα isoform specificity. Our comparative analysis using publicly-available human ChIP-seq data based on ENCODE-derived ESRRA binding sites (from normoxic K562 cells) revealed a preferential association at HIF-2α binding sites in hypoxic HepG2 cells but rather at HIF-1α binding sites in hypoxic HKC8 and RCC4 cells (Figure S3). Consistently, motif analysis of the proximal regulatory regions of HIF-α target genes in primary human endothelial cells upon overexpression of HIF-1α and HIF-2α showed that the ESRRA motif is enriched in sites flanking the transcription starting site. Downes and colleagues also suggested that HIF-2α may regulate a subset of its target genes either directly through mechanisms independent of its DNA binding or through indirect regulation of other transcription factors [42]. Furthermore, a recent study demonstrated that in mouse proximal convoluted tubule and proximal straight tubule kidney cells, Esrra motifs were enriched and positively correlated with proximal tubule differentiation [19]. Additionally, Dhillon and colleagues showed that the absence of Esrra resulted in increased fibrosis and altered mitochondrial function after folic acid-induced nephropathy. Finally, they could demonstrate that ESRRA levels were significantly lowered and strongly correlated with reduced eGFR and increased kidney fibrosis in human samples of CKD patients [19].

ESRRs display similar structures and share common regulatory functions on metabolic gene programs, suggesting potentially redundant functions [43]. Our RNAseq results show that *Esrra* and *Esrrg* are both expressed in mCCD_cl1_ cells. However, even though Esrrg was previously reported to be expressed in kidney tubules [44], the levels of *Esrrg* were dramatically lower than *Esrra* in our RNAseq data with normalized counts almost 4 times lower under normoxic conditions and more than 33 times lower under hypoxic conditions. Moreover, under low oxygen levels, *Esrrg* is strongly down-regulated with a remaining expression of about 7%. Furthermore, *Esrrb* was not detectable in our cells in either condition. In addition, in our Esrra knockdown experiments, two independent shRNA targeting sequences were used, displaying similar effects (Figure 5). Taken together, our results strongly suggest that the reduced expressions of *Egln3* and *Serpine1* under hypoxic conditions are mainly mediated by Esrra.

Collectively, we have shown in this study that mCCD_cl1_ cells respond to hypoxia and promote the expression of HIF target genes under hypoxic conditions through HIF-1. Our experimental data suggest Esrra as a potential co-factor of hypoxia-mediated transcriptional regulation at least in mCCD cells. Future research shall address whether Esrra represents a broader co-factor of the HIF-mediated transcriptional response and if Esrra could function as a specific transcriptional regulator of hypoxia-dependent expression in other cell lines as well as in vivo. If validated, it would be of major interest to understand the precise mechanism for a putative isoform selectivity function.

## 4. Materials and Methods

### 4.1. mCCDcl1 Cell Culture

mCCD_cl1_ cells were maintained at 37 °C and 5% CO_2_ in DMEM/F-12 medium (Thermofisher, 31331093, Waltham, MA, USA), supplemented with 5 µg/mL insulin (Sigma, I-1882, St. Louis, MO, USA), 50 nM dexamethasone (Sigma, D-8893), 60 nM selenium (Sigma, S-9133), 5 µg/mL transferrin (Sigma, T-1428), 1 nM triiodothyronine (Sigma, T-5516), 5 ng/mL mouse EGF (Sigma, E-4127), 100 µg/mL penicillin/streptomycin glutamine (Thermofisher, 10378016), and 2% decomplemented fetal bovine serum (Thermofisher, 10270106). For hypoxic culture, the medium was freshly replaced, and cells were maintained for the indicated time at 0.2% O_2_ and 5% CO_2_ in a gas-controlled glove box (InvivO2 400, Baker Ruskinn, Bridgend, UK). Cellular viability was routinely analyzed by trypan blue exclusion using the TC20 automated cell counter (Biorad, Hercules, CA, USA).

### 4.2. Generation of Stable Knockdown Cell Lines

Expression vectors encoding the short hairpin RNA (shRNA) sequences targeting mouse Hif-1α and Esrra in a pLKO.1-puro plasmid were purchased from Sigma-Aldrich (shHif-1α-1: order number TRCN0000232221; shHif-1α-2: order number TRCN0000232222; shHif-1α-3: order number TRCN0000232223; shEsrra-1: order number TRCN0000238023; shEsrra-2: order number TRCN0000238024). Control cells (shCtrl) were transfected with a non-targeting control shRNA under the control of a U6 promoter in a pKLO.1 puromycin resistance vector (Sigma-Aldrich) as described previously [45]. Viral particles were produced in HEK293T cells by co-transfection of the respective transfer vector (3 μg) with the packaging plasmids pLP1 (4.2 μg), pLP2 (2 μg), and pVSV-G (2.8 μg, all from Invitrogen) using CaCl_2_ transfection as described before [46]. mCCD_cl1_ cells were transduced with lentiviral-pseudotyped particles, and cell pools were cultured in DMEM supplemented with 10% FBS and 1% penicillin/streptomycin with the appropriate antibiotic for selection.

### 4.3. RNA Preparation and RT-qPCR

Total RNA was extracted using the RNeasy Mini Kit (Qiagen, 74106, Hilden, Germany) according to the manufacturer’s instructions. cDNA was prepared from RNA extracts using the PrimeScript RT Reagent Kit (Takara, RR037A, Kusatsu shi, Japan) according to the manufacturer’s instructions. The expression of selected genes was analyzed by quantitative real-time PCR performed on a CFX96 C1000 Thermocycler (BioRad) using Kapa SYBRgreen PCR master mix (Sigma, KK4617) as previously described [47]. The expression of the different analyzed genes was normalized to the expression of β-actin. The primer sequences are given in Table 1.

### 4.4. RNA Sequencing

mRNA was extracted from mouse mCDD_cl1_ cells in triplicate for each treatment (hypoxia vs. normoxia) using the RNeasy Mini Kit (Qiagen, 74106) according to the manufacturer’s instructions. Library preparation was performed for each sample with the TruSeq total RNA kit with the removal of rRNA according to the manufacturer’s protocols. mRNAs were sequenced at 100 bp paired-end on the NovaSeq Illumina platform of the University of Bern. RNA reads were deposited at EBI-SRA with accession number PRJEB53226 (ERP138014).

### 4.5. Quality Control and Cleaning

The reads were QC, with FastQC v0.11.9 [48] and MultiQC v1.8 [49] revealing no particular issue. The reads were filtered for low quality and trimmed with fastp [50], keeping only reads with a minimum length of 100 bp (--trim_poly_g --trim_front1=0 --trim_front2=0 -l 100).

### 4.6. Remapping and Counting

The cleaned reads were aligned to the reference genome GRCm38.p6 (ENSEMBL [51]) with STAR v2.7.3a [52] (--quantMode GeneCounts --outFilterMultimapNmax 2). STAR reported between 80% and 85% uniquely mapped reads to the mouse reference genome, which is within the expected range.

### 4.7. Differential Gene Expression Analysis

Raw counts were transformed with EdgeR with a minimum value of 0.5 CPM per library. The trimmed mean of M-values was used to normalize the counts, dispersion was estimated with locfit, and glmQLFit was applied for DE test [53,54,55]. DESeq2 identified a total of 3086 genes (FDR 0.1, logFC < −1 or > 1, adjusted *p*-value < 0.05) as differentially expressed between the two conditions with 1679 genes up-regulated and 1407 genes down-regulated in the hypoxia-treated samples.

### 4.8. Enrichement Pathway Cluster Analysis k-Means

GO biological process enrichment pathway analysis was performed on 4 different clusters based on their expression pattern, and the adjusted *p*-value was calculated for each pathway [56].

### 4.9. Kyoto Encyclopedia Genes and Genomes (KEGG) Pathway Analysis

HIF-1 related pathway analysis was performed between normoxic and hypoxic conditions and represented with the Pathview tool using KEGG integrative databases [57,58].

### 4.10. Gene Set Enrichment Analysis (GSEA)

GSEA was performed on normalized counts with the GSEA software v4.2.3 [59,60]. An FDR of 0.1 was applied. The molecular signatures database (MSigDB) enrichment hallmarks were assessed [61,62]. Hallmark hypoxia was positively correlated with the treatment (hypoxic condition) with an enrichment score (ES) of 0.6350684 and a normalized enrichment score (NES) of 2.8968365. Finally, the heatmap of the geneset members was generated based on the rank-ordered list metric score.

### 4.11. Sample Preparation and Immunoblotting

After 24 h under normoxic or hypoxic (0.2% O_2_) conditions, cells were lysed with NP-40 lysis buffer (10 mM Tris-HCl pH 8.0, 400 mM NaCl, 1 mM EDTA pH 8.0, 0.1% NP-40, 2 μg/mL aprotinin, 4 μg/mL leupeptin, 2 μg/mL pepstatin, and 1 mM PMSF) and centrifuged for 15 min at 13,000 rpm and 4 °C. Protein quantification was performed by Lowry assay. Samples denatured in SDS-PAGE sample buffer were separated on SDS acrylamide gels and transferred onto PVDF membranes as described before [63] The blots were incubated with primary antibodies (anti-HIF-1α, Cayman, 10006421-1ea, anti-β-actin, Sigma, SP124) overnight, and for 1 h with horseradish-conjugated secondary antibodies (anti-mouse IgG HRP, Sigma, GENA931-1ML; anti-rabbit IgG HRP, Sigma, GENA934-1ML). The signal was revealed using ECL Prime (Amersham, GERPN2232) on a C-DiGit^®^ Western blot scanner (LI-COR Biosciences, Lincoln, NE, USA), and quantified using ImageStudioTM program (LI-COR Biosciences). Uncropped immunoblots are provided in Supplementary Materials.

### 4.12. Comparative ChIP-Sequencing Analysis in Human Cell Lines

An in-house algorithm was developed to compare ChIP-seq data sets across the genome. The following public ChIP-seq data sets were employed, available at GEO: GSE91805 (ESRRA in K562) and GSE120885 (HIF-1α, HIF-2α, and HIF-β in RCC4, HepG2, and HKC8). Venn diagrams were generated using PNNL and the Venn-Diagram-Plotter GitHub repository software (https://github.com/PNNL-Comp-Mass-Spec/Venn-Diagram-Plotter/blob/master/Readme.md accessed on 31 May 2022) [64].

### 4.13. Statistical Analysis

All values in the figures are presented as the mean ± standard error of the mean (SEM). Differences in means between groups were analyzed with one-way ANOVA followed by Tukey’s post-hoc test. All statistics were performed with GraphPad Prism software 7.1. Values of *p* ≤ 0.05 were considered statistically significant.

## Figures and Tables

**Table 1:** List of mouse SYBRgreen primers used throughout the study.

Gene	Forward Primer (5′-3′)	Reverse Primer (5′-3′)
*Hif-1α*	ACA AGT CAC CAC AGG ACA G	AGG GAG AAA ATC AAG TCG
*Egln1*	GCA ACG GAA CAG GCT ATG TC	CTC GCT CAT CTG CAT CAA AA
*Egln3*	CAA CTT CCT CCT GTC CCT CA	GGC TGG ACT TCA TGT GGA TT
*Glut1*	TCT CTG TCG GCC TCT TTG TT	GCA GAA GGG CAA CAG GAT AC
*Vegfa*	TCC CAC AGG TGT CCC GGC AA	TCT CTG CCT CCG TGA GGG GC
*Pag1*	GTG GCC ATG TTC TTC CTC AT	GGA AGG CAC ATT CAT CAG GT
*Serpine1*	GAC ACC CTC AGC ATG TTC ATC	AGG GTT GCA CTA AAC ATG TCA G
*Soat2*	GGC TGT ACA GCT ATG TGT ATC AAG	TTA GGG ATG GCA GGA CCA AGA
*Fosl1*	CGC AAG CTC AGG CAC AGA	AAT GAG GCT GCA CCA TCC A
*Rhcg*	GCG CTG TAG GCT TCA ACT TC	GGC TGA CCT TGC CTA GAA CT
*HSD11b2*	GAG GGG ACG TAT TGT GAC CG	TCA CAT TAG TCA CTG CAT CTG TC
*Scnn1a*	CGG AGT TGC TAA ACT CAA CAT C	TGG AGA CCA GTA CCG GCT
*Scnn1b*	GTC ATC GGA ACT TCA CGC CTA T	TCC TCC TGA CCG ATG TCC AG
*Esrra*	AGTGCTCAGCTCTCTACCCA	TACTCGATGCTCCCCTGGAT
*β-actin*	GAG CGT GGT TAC AGC TTC AC	GGC ATA GAG GTC TTT ACG GAT G

## Data Availability

Not applicable.

## Supplementary Materials

Additional supporting information can be downloaded at: https://www.mdpi.com/article/10.3390/ijms23137262/s1.
