# Supplementary material for: Analysis of the Hypoxic Response in a Mouse Cortical Collecting Duct-Derived Cell Line Suggests That Esrra Is Partially Involved in Hif1α-Mediated Hypoxia-Inducible Gene Expression in mCCDcl1 Cells
Source: Int J Mol Sci. 2022 Jun 30;23(13):7262. doi: 10.3390/ijms23137262 (PMC9267015; doi:10.3390/ijms23137262)
Supplement: Supplementary file 1 [file ijms-23-07262-s001.zip › ijms-1774981-supplementary.pdf]

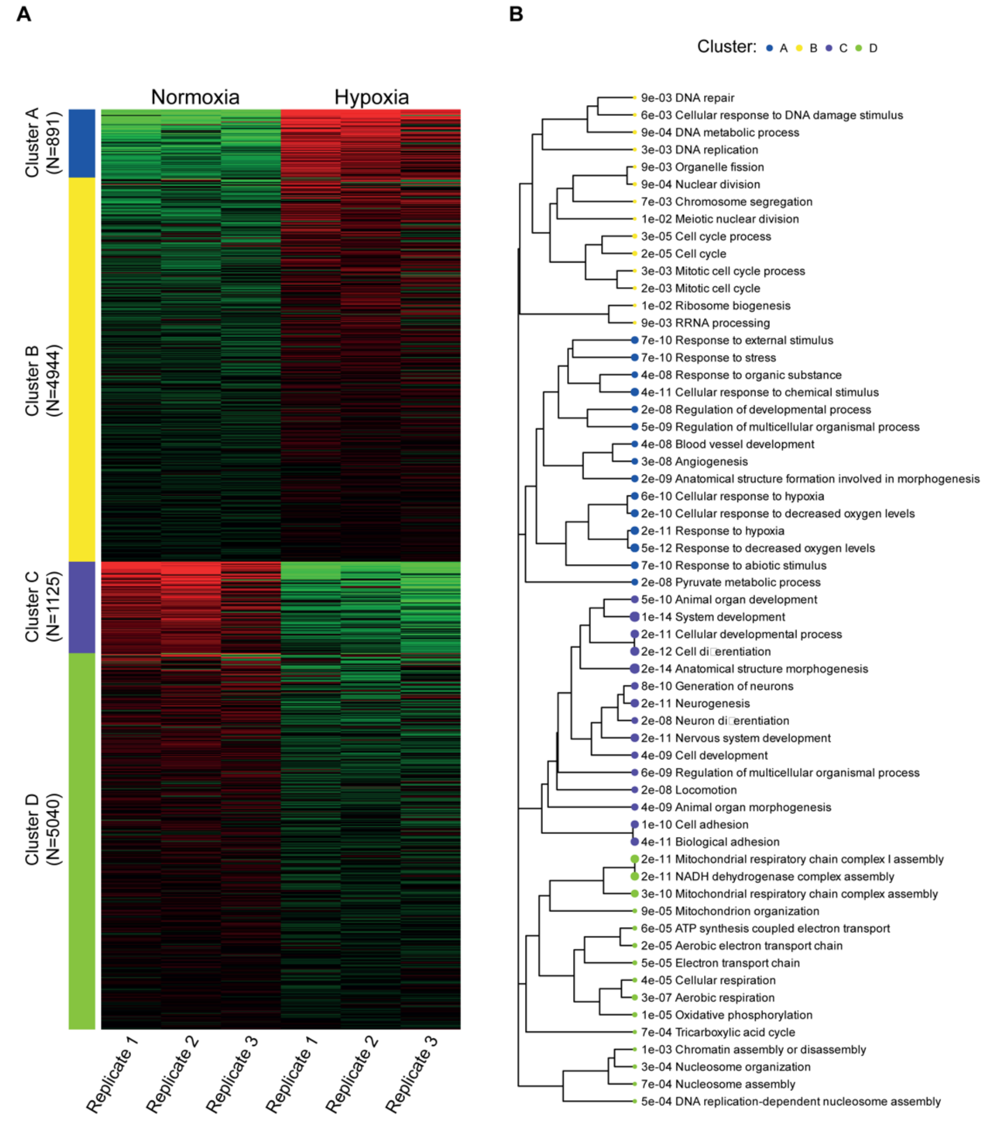

Figure S1. k-Means enrichment representation of 4 different clusters analysed according to GO Biological Process. (**A**) Heatmap of the different clusters with the number of genes indicated for each. (**B**) Enriched pathways for each cluster are displayed along with the adjusted *p*-value.

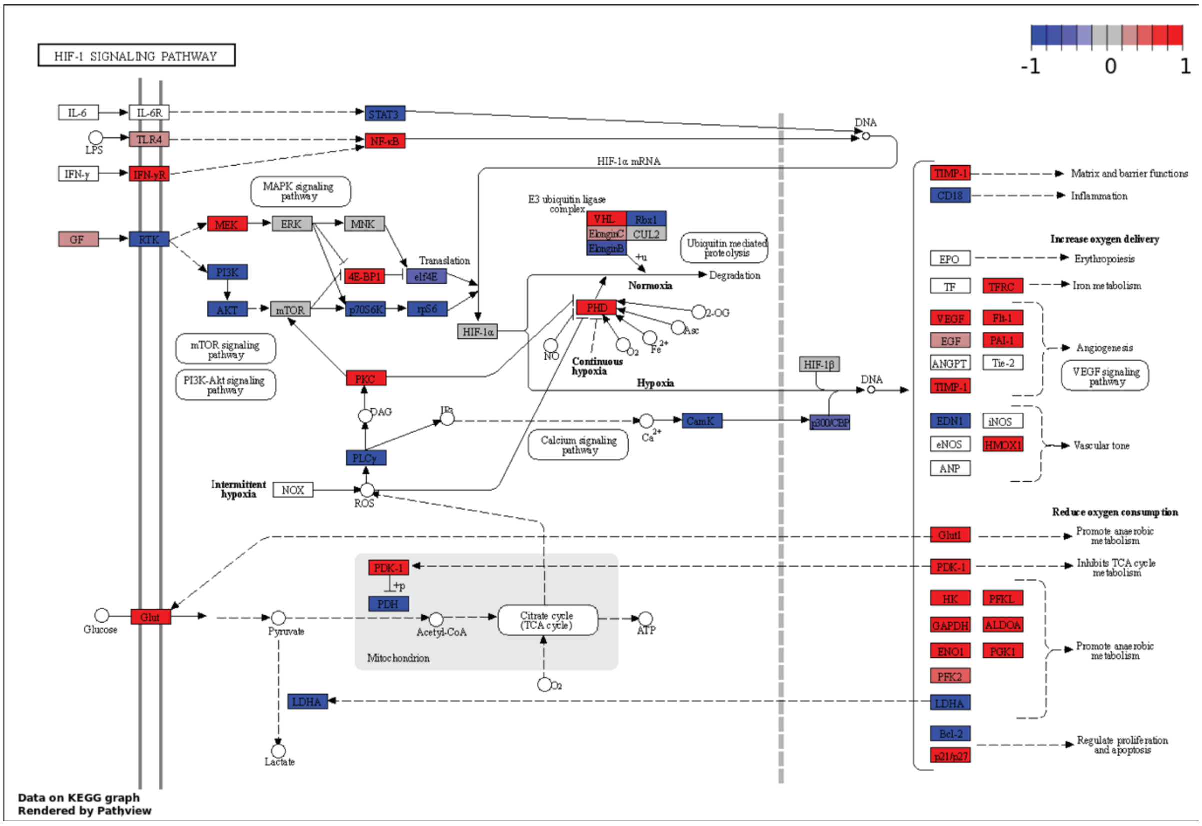

Figure S2. Specific Hif-1α pathway gene enrichment in mCCD$_{cl1}$ cells. Significantly up- and down-regulated genes are indicated in red and blue, respectively.

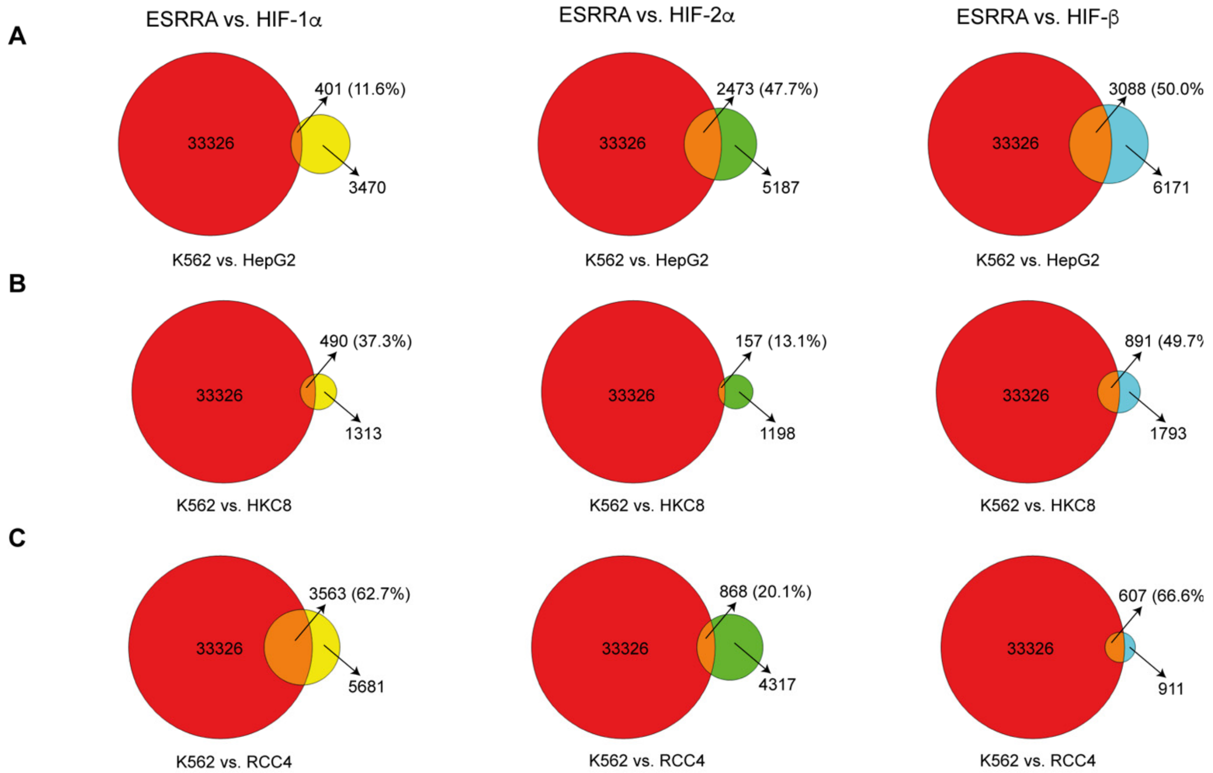

Figure S3. Venn diagrams of ChIP-seq analysis show strong binding of HIF-α/β along with ESRRA. Binding peaks positions of ESRRA in K562 were compared to HIF-α/β in (**A**) HepG2, (**B**) HKC8 and (**C**) RCC4 cell lines and the number of binding sites are indicated as well as the percentage of overlapping sites (with a margin error of 10%).

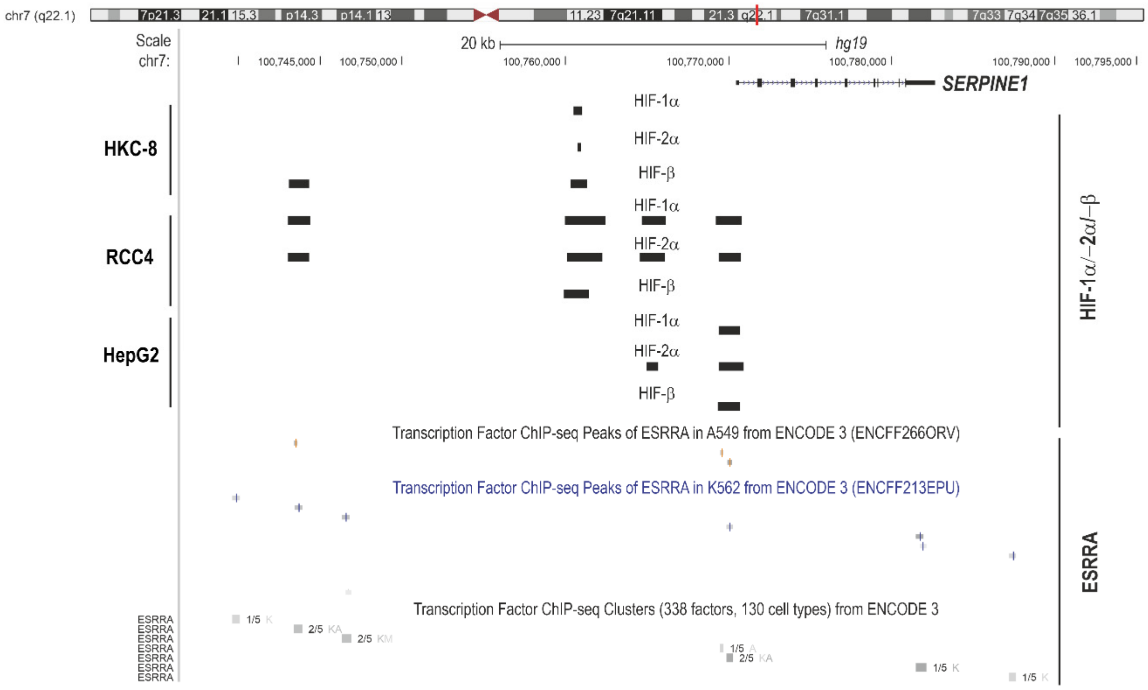

Figure S4. UCSC Genome Browser output (*hg19*) of the *SERPINE1 (PAI1)* genomic region. HIF-1α, HIF-2α and HIF-β ChIP-seq data of hypoxic HKC-8, RCC4 and HepG2 cell lines are indicated. For ESSRA, ChIP-seq data in normoxic A549 and K562 cell lines as well as additional cell lines from the ENCODE integrated regulation track are displayed.

Original uncropped immunoblots

Fig. 3G IB Hif-1α and β-actin

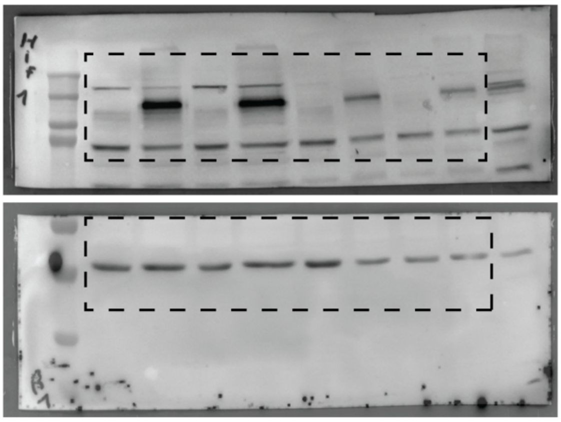

Fig. 4A IB Hif-1α and β-actin

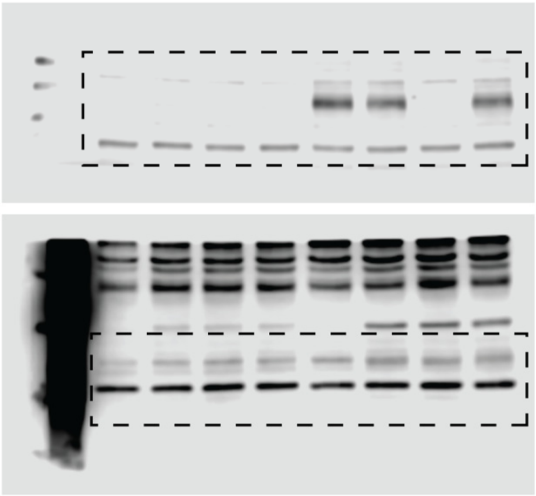

Fig. 5B IB Hif-1α and β-actin

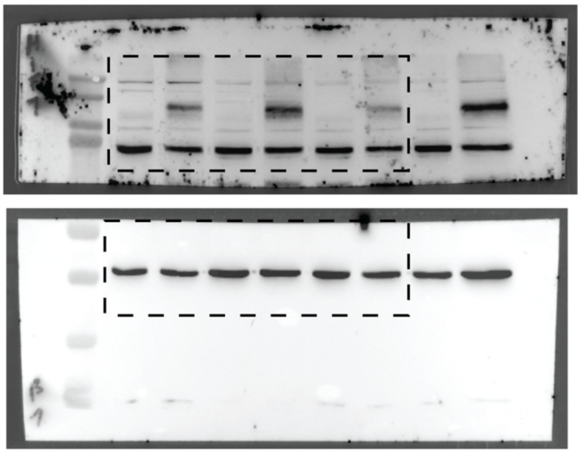

Uncropped immunoblots for figures 3G, 4A, and 5B.
